# The pseudogene PRELID1P6 promotes glioma progression via the hnHNPH1-Akt/mTOR axis

**DOI:** 10.1038/s41388-021-01854-x

**Published:** 2021-06-09

**Authors:** Shaoyan Xi, Haiping Cai, Jiabin Lu, Yu Zhang, Yanjiao Yu, Furong Chen, Qitao Huang, Fang Wang, Zhongping Chen

**Affiliations:** 1 Department of Pathology, Sun Yat-sen University Cancer Center, State Key Laboratory of Oncology in South China, Collaborative Innovation Center for Cancer Medicine, Guangzhou, People’s Republic of China; 2 Department of Neurosurgery/Neuro-oncology, Sun Yat-sen University Cancer Center, State Key Laboratory of Oncology in South China, Collaborative Innovation Center for Cancer Medicine, Guangzhou, People’s Republic of China; 3 Department of Molecular Diagnostics, Sun Yat-sen University Cancer Center, Collaborative Innovation Center for Cancer Medicine, Guangzhou, People’s Republic of China

**Keywords:** Cancer prevention, CNS cancer

## Abstract

Research over the past decade has suggested important roles for pseudogenes in glioma. This study aimed to show that pseudogene PRELI domain-containing 1 pseudogene 6 (PRELID1P6) promotes glioma progression. Aberrant expression of genes was screened using The Cancer Genome Atlas database. We found that mRNA level of PRELID1P6 was highly upregulated in glioma and was associated with a shorter survival time. Functional studies showed that the knockdown of PRELID1P6 decreased cell proliferation, sphere formation, and clone formation ability and blocked the cell cycle transition at G0/G1, while overexpression of PRELID1P6 had the opposite effects. Mechanistically, knockdown of PRELID1P6 changed the cellular localization of heterogeneous nuclear ribonucleoprotein H1 (hnRNPH1) from nucleus to cytoplasm, which promoted ubiquitin-mediated degradation of hnRNPH1. RNA-sequence and gene set enrichment analysis suggested that knockdown of PRELID1P6 regulates the apoptosis signaling pathway. Western blotting showed that PRELID1P6 increased TRF2 expression by hnRNPH1-mediated alternative splicing effect and activated the Akt/mTOR pathway. Furthermore, Akt inhibitor MK2206 treatment reversed the oncogenic function of PRELID1P6. PRELID1P6 was also found to be negatively regulated by miR-1825. Our result showed that PRELID1P6 promotes glioma progression through the hnHNPH1-Akt/mTOR pathway. These findings shed new light on the important role of PRELID1P6 as a novel oncogene for glioma.

## Introduction

Gliomas are the most common type of primary malignant brain tumor in adults. They are associated with a short survival, and direct repercussions on the quality of life and cognitive functions [1, 2]. Even with standard therapy, including surgical resection, radiotherapy, and chemotherapy, the prognosis for glioma patients remains poor [3, 4]. Further advancements in technologies to identify new biomarkers to improve the understanding of the regulation of cell processes and treatment of glioma are needed.

Whole-genome sequencing has revealed that most of the human genome is transcribed, while only a small part contains protein-coding genes [5]. The remainder of the genome encodes mainly noncoding RNAs. Mounting evidence has indicated that noncoding RNAs are involved in various cellular processes, including the regulation of epigenetic signatures and gene expression [6–10]. Similarly, noncoding RNAs were also reported to be involved in the pathogenesis of cancer with diverse functions and mechanisms [11, 12]. Pseudogenes, noncoding genes, were first introduced in 1977 and were considered junk genes for a long time [13]. With advancements in next-generation sequencing, pseudogenes were found to play critical roles in transcriptional and posttranscriptional regulation in cancer cells [14]. For example, the pseudogene OCT4-pg4 was reported to function as a competing endogenous RNA (ceRNA) in liver cancer and is significantly related to the patients’ prognosis [15]. One study showed that transcriptional or genomic aberrations of the BRAF pseudogene frequently occurred in B-cell lymphomas [16]. Similarly, ferritin heavy polypeptide 1 pseudogene 3 was reported to be upregulated in glioma and promoted glioma cell proliferation via the downstream mechanism of the miR-224-5p/TPD52 axis [17]. Therefore, research on pseudogenes and deeper understanding of their biological functions are important to decipher the etiology of cancer, and facilitate new approaches for cancer screening, prevention, and treatment.

In recent years, pseudogenes have been identified to be involved in cancer progression, but the underlying mechanism remains unclear. In this study, we found that the pseudogene PRELI domain-containing 1 pseudogene 6 (PRELID1P6) plays a pivotal role in glioma cell proliferation by enhancing heterogeneous nuclear ribonucleoprotein H1 (hnRNPH1) stability. PRELID1P6 was found to be highly expressed in both glioma cells and tissues, and was correlated with a shorter survival of glioma patients. PRELID1P6-promoted glioma proliferation in vitro and in vivo. Mechanistically, PRELID1P6 inhibits the ubiquitin-mediated degradation of hnRNPH1, thus promoting glioma cell proliferation by the Akt/mTOR signaling pathway. In addition, PRELID1P6 is negatively regulated by miR-1825. Our study suggests that the pseudogene PRELID1P6 may be a biomarker and therapeutic target for glioma treatment.

## Results

### PRELID1P6 is highly expressed in glioma, and the upregulation of PRELID1P6 predicts a poor outcome

**Fig. 1:**
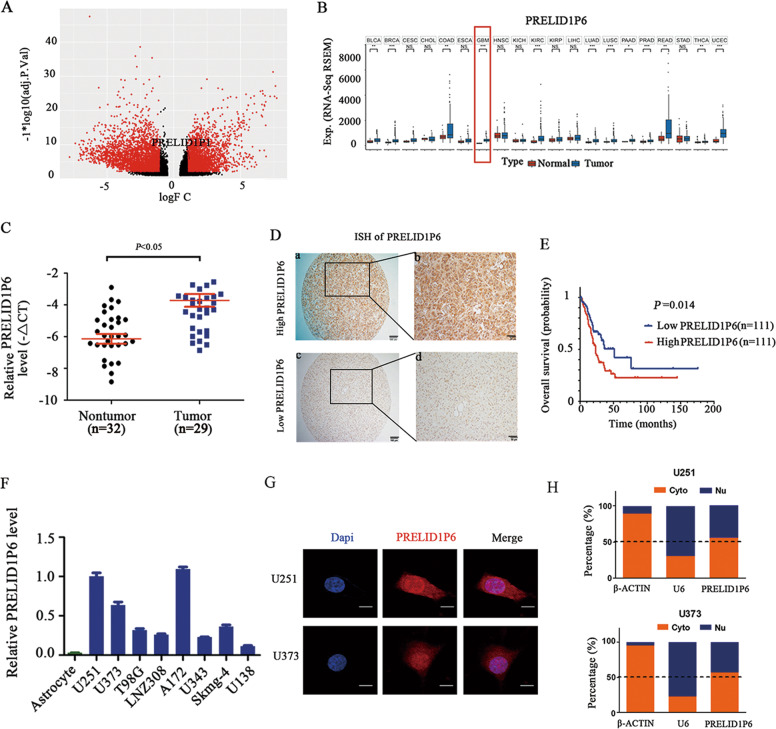
PRELID1P6 is highly expressed in glioma and its upregulation predicts a poor outcome. **A** Aberrant gene expression in glioma as shown in the TCGA database. **B** The expression of PRELID1P6 was significantly higher in tumors than in their corresponding normal tissues, including glioblastoma. (BLCA bladder urothelial carcinoma, BRCA breast invasive carcinoma, CESC cervical squamous cell carcinoma and endocervical adenocarcinoma, CHOL cholangiocarcinoma, COAD colon adenocarcinoma, ESCA esophageal carcinoma, GBM glioblastoma multiforme, HNSCC head and neck squamous cell carcinoma, KICH kidney chromophobe, KIRC kidney renal clear cell carcinoma, KIRP kidney renal papillary cell carcinoma, LIHC liver hepatocellular carcinoma, LUAD lung adenocarcinoma, LUSC lung squamous cell carcinoma, PAAD pancreatic adenocarcinoma, PRAD prostate adenocarcinoma, READ rectal adenocarcinoma, STAD stomach adenocarcinoma, THCA thyroid carcinoma, UCEC uterine corpus endometrial carcinoma). **C** mRNA level of PRELID1P6 was upregulated in human glioma tissues compared with that in adjacent normal tissues, as confirmed by qRT-PCR (nontumor: *n* = 32; tumor: *n* = 29; seven data points were excluded because they were outside the axis limits). **D** PRELID1P6 expression was detected by ISH in paraffin tissues. **A**, **C**: scale bar = 100 μm; **B**, **D**: scale bar = 50 μm. **E** The overall survival curves of patients with low or high levels of PRELID1P6 were generated using the Kaplan‒Meier method (*P* = 0.014). **F** PRELID1P6 was upregulated in human glioma cell lines and downregulated in normal human astrocytes, as confirmed by qRT-PCR. **G** PRELID1P6 accumulated both the cytoplasm and nucleus, as observed by FISH. Scale bar = 20 μm. **H** PRELID1P6 accumulated both in cytoplasm and nucleus, as detected by subcellular fractionation analysis.

To identify oncogenic long noncoding RNAs (lncRNAs) that significantly affect glioma progression, we first identified lncRNAs that were highly upregulated in glioma tissues compared with that in paired adjacent normal tissues from The Cancer Genome Atlas (TCGA) database (Fig. 1A). We found that the mRNA level of PRELID1P6 was significantly higher in glioma tissues than that in adjacent normal tissues (Fig. 1B), a finding that was further confirmed by a cohort containing 34 cases of paired fresh glioma and adjacent nontumorous brain tissues from Sun Yat-sen University Cancer Center (SYSUCC; seven data points were excluded because they were outside the axis limits, Fig. 1C). Bioinformatics analysis show that PRELID1P6 is located at chromosome 2p15 and has weak protein-coding function (the PRELID1P6 level were detected at mRNA level throughout our study; Fig. S1A, B). In addition, the PRELID1P6 level was detected on a tissue microarray containing 222 patient samples by the in situ hybridization (ISH) assay and Kaplan‒Meier analyses showed that high expression of PRELID1P6 predicted a shorter survival (Fig. 1D, E). Univariate Cox regression analysis revealed that younger age (≤50 years; *P* = 0.005), low tumor grade (grade I to II; *P* < 0.001), IDH mutation (*P* = 0.02), and PRELID1P6 downregulation (*P* < 0.001) were significantly related to better overall survival (Table 1). Multivariate Cox regression analysis indicated that the downregulation of PRELID1P6 and IDH mutation were independent prognostic factors for glioma patients (*P* < 0.001 and *P* = 0.024, respectively; Table 1). Next, we detected the PRELID1P6 level in eight glioma cell lines and a human immortalized normal astrocyte cell line, and found that the level was significantly higher in tumor cell lines than in the normal astrocytes (Fig. 1F). To identify the subcellular localization of PRELID1P6, we detected the PRELID1P6 level in cytoplasmic and nuclear fractions by fluorescence in situ hybridization (FISH) and qRT-PCR. FISH result showed that PRELID1P6 was located both in the cytoplasm and nucleus (Fig. 1G). MRNA level of PRELID1P6 in cytoplasm and nucleus were detected by qRT-PCR using Cytoplasmic and Nuclear RNA Purification Kit (Fig. 1H). Collectively, our results suggested that PRELID1P6 is highly expressed in glioma and high expression of PRELID1P6 predicts a shorter survival of glioma patients.

**Table 1:** Cox proportional hazards model to analyze OS in glioma patients.

Clinicopathologic features	Univariate analysis			Multivariate analysis		
	Risk ratio	95% CI	*P*	Risk ratio	95% CI	*P*
Gender	1.2	0.786–1.833	0.399			
1p19q co-deletion	1.283	0.927–1.776	0.132			
Age (years)	1.791	1.196–2.681	0.005*	0.768	0.368-1.604	0.483
Grade	2.772	1.641–4.684	<0.001*	1.991	0.765-5.182	0.158
IDH1 mutation	0.261	0.113–0.602	0.002*	0.349	0.140-0.871	0.024*
PRELID1P6 expression	2.680	1.730–4.150	<0.001*	3.554	1.742-7.252	<0.001*

*CI* confidence interval.

*Bold values indicates statistically significant *P* < 0.05.

### PRELID1P6 promotes glioma cell proliferation and inhibits apoptosis

**Fig. 2:**
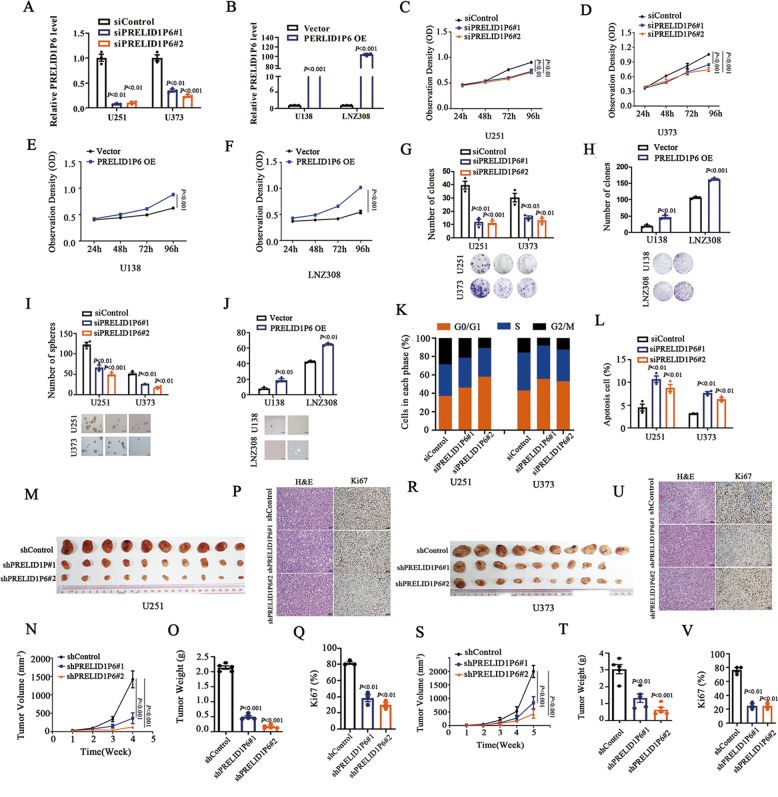
Knockdown of PRELID1P6 significantly decreases glioma cell proliferation in vitro and in vivo. **A**, **B** The mRNA levels of PRELID1P6 were significantly downregulated in U251 and U373, or upregulated in U138 and LNZ308, as confirmed by qRT-PCR. GAPDH was used as the control. **C**, **D** Knockdown of PRELID1P6 significantly reduced U251 and U373 cell proliferation. The data are shown as means ± s.e.m. from three experiments. **E**, **F** Overexpression of PRELID1P6 significantly promoted U138 and LNZ308 cell proliferation. The data are shown as means ± s.e.m. from three experiments. **G** Knockdown of PRELID1P6 inhibited the clone formation ability of glioma cells compared with control cells. **H** Overexpression of PRELID1P6 promoted the clone formation ability of glioma cells compared with control cells. **I** Knockdown of PRELID1P6 inhibited the sphere formation ability of glioma cells. Scale bar = 100 μm. **J** Overexpression of PRELID1P6 promoted the sphere formation ability of glioma cells compared with control cells. Scale bar = 100 μm. **K** Knockdown of PRELID1P6 blocked the cell cycle transition at G0/G1 in U251 (left) and U373 cells (right). **L** Knockdown of PRELID1P6 significantly increased the cell apoptosis of U251 (left) and U373 cells (right). **M**–**O** Xenograft tumor formation of U251 cells with PRELID1P6 knockdown in nude mice. The growth rates and tumor weights were significantly lower than those of the control group. **P**, **Q** H&E staining and IHC staining for Ki67 confirmed the downregulation of cellularity and Ki67 in xenograft tumors from the PRELID1P6 knockdown group. **R**–**T** Xenograft tumor formation of U373 cells with PRELID1P6 knockdown in nude mice. The growth rates and tumor weights were significantly lower than those in the control group. **U**, **V** H&E staining and IHC staining for Ki67 confirmed the downregulation of cellularity and Ki67 in xenograft tumors from the PRELID1P6 knockdown group. Scale bar = 50 μm.

According to the expression of PRELID1P6 in the glioma cell lines (Fig. 1F), two shRNAs targeting PRELID1P6 and a control sequence were transfected into U251 and U373 cell lines, while a lentiviral PRELID1P6 construct and control were stably transfected into U138 and LNZ308 cell lines. qRT-PCR confirmed that the expression of PRELID1P6 was significantly downregulated or upregulated in glioma cell lines (Fig. 2A, B). We next examined the effects of PRELID1P6 on cell phenotypes, and found that the knockdown of PRELID1P6 in U251 and U373 cell lines substantially reduced the cell proliferation (Fig. 2C, D), while PRELID1P6 overexpression promoted U138 and LNZ308 cell proliferation (Fig. 2E, F). The clone and sphere formation abilities in both U251 and U373 cell lines were markedly suppressed after PRELID1P6 knockdown, while PRELID1P6 overexpression promoted the clone and sphere formation abilities in U138 and LNZ308 cells (Fig. 2G‒J). Furthermore, flow cytometry analysis indicated that the knockdown of PRELID1P6 blocked the G1/S cell cycle transition and increased the rate of apoptosis (Fig. 2K, L).

Because the knockdown of PRELID1P6 inhibited glioma cell proliferation and induced cell apoptosis in vitro, we evaluated the impact of PRELID1P6 on glioma cell tumorigenesis in vivo. Subcutaneous tumor xenografting assays of U251 and U373 in nude mice showed that the volume and weight of xenograft tumors from the PRELID1P6 knockdown groups were significantly lower than their corresponding controls. H&E and Ki67 immunohistochemical (IHC) staining confirmed the downregulation of cellularity and Ki67 in xenograft tumors from the PRELID1P6 knockdown groups (Fig. 2M‒V). In addition, xenograft tumors formed by PRELID1P6-overexpressed cells were significantly larger than those of their corresponding controls (Fig. S2A‒C). Together, our data suggested that PRELID1P6 promotes cell proliferation and inhibits glioma cell apoptosis.

### PRELID1P6 interacts with hnRNPH1 and prevents the ubiquitination (Ub) degradation of hnRNPH1

**Fig. 3:**
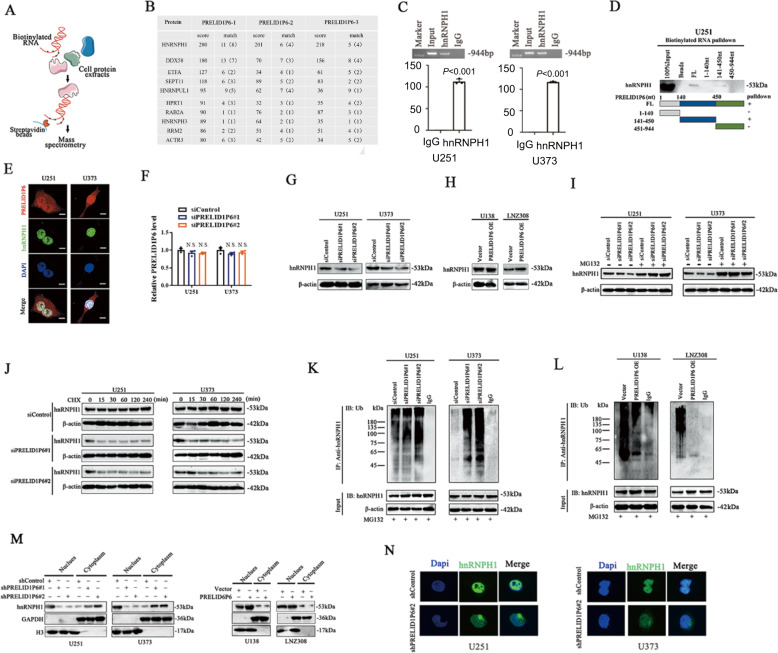
PRELID1P6 interacts with hnRNPH1 and prevents ubiquitination (Ub)-mediated degradation of hnRNPH1. **A** Schematic representation of the RNA pull-down assay to identify proteins associated with PRELID1P6. **B** Mass spectrometry identified the PRELID1P6–protein complex and showed that hnRNPH1 was the major protein in the complex. **C** RIP assays showed that hnRNPH1 interacted with PRELID1P6. **D** Nucleotides sites at 141–450 of PRELID1P6 were sufficient to interact with hnRNPH1 protein. FL full-length sequence. **E** Representative images of costaining PRELID1P6 (red) and hnRNPH1 (green) in U251 and U373 cells by the combination of RNA FISH and immunofluorescence. Nuclei were stained with DAPI (blue). Scale bar = 20 μm. **F** Knockdown of PRELID1P6 did not decrease the mRNA levels of hnRNPH1 in U251 and U373, as detected by qRT-PCR. **G** Knockdown of PRELID1P6 decreased the protein expression of hnRNPH1 in glioma cells U251 and U373, as detected by western blotting. **H** Overexpression of PRELID1P6 increased the protein expression of hnRNPH1 in glioma cells U138 and LNZ308, as detected by western blotting. **I** U251 and U373 cells with PRELID1P6 knockdown or control cells were treated with MG-132 (5 μM) or vehicle for 24 h. The protein level of hnRNPH1 was increased after MG-132 treatment. **J** U251 and U373 cells with PRELID1P6 knockdown or control cells were treated with 20 μg/mL of CHX for 0, 15, 30, 60, 120, and 240 min. The protein level of hnRNPH1 levels was analyzed by western blotting. **K** U251 and U373 cells with PRELID1P6 knockdown. **L** U138 and LNZ308 with PRELID1P6 overexpression were treated with MG-132 (5 μM) for 24 h. Cell lysates were immunoprecipitated (IP) with either control IgG or antibody against hnRNPH1 and then were analyzed by immunoblotting with a ubiquitin (Ub)-specific antibody. Bottom panel, input of cell lysates. **M**, **N** The protein of hnRNPH1 increase in cytoplasm, while decrease in nucleus after PRELID1P6 knockdown in U251 and U373 glioma cells detected by western blot or IF (H3 used as the nucleus control, GAPDH used as the cytoplasm control).

As reported previously, noncoding RNA may function through interaction with proteins [18]. To dissect the molecular mechanisms underlying PRELID1P6-mediated cell proliferation and apoptosis, we tried to identify PRELID1P6-associated proteins using RNA pull-down assays followed by mass spectrometry (Fig. 3A). Mass spectrometry analysis revealed many proteins that could interact with PRELID1P6, while the most abundant protein among them was hnRNPH1 (Fig. 3B and Table S1). The interaction between hnRNPH1 and PRELID1P6 was further validated by RNA immunoprecipitation (RIP) assays in U251 and U373 cells (Fig. 3C). We excluded the possibility of interaction between PRELID1P6 and other RNA-binding proteins (RBPs) like DDX58, hnRNPUL1, and hnRNPH3 (Fig. S3A‒C). To map the PRELID1P6 functional motifs corresponding to hnRNPH1 binding, the secondary structures of PRELID1P6 were predicted by RNAfold (Fig. S4). We then conducted RNA pull-down assays using a series of truncated PRELID1P6 fragments and found that nucleotides 141–450 of PRELID1P6 were sufficient to interact with hnRNPH1 protein (Fig. 3D). Furthermore, RNA FISH combined with immunofluorescence (IF) demonstrated the colocalization of PRELID1P6 and hnRNPH1 in U251 and U373 cells in the nucleus, implying that the PRELID1P6–hnRNPH1 complex may play a role in the nucleus (Fig. 3E).

Next, we tried to explore the interacting mechanism between PRELID1P6 and hnRNPH1. We found that the hnRNPH1 mRNA level did not change after PRELID1P6 knockdown in U251 and U373 cells (Fig. 3F), while the protein level of hnRNPH1 was decreased in PRELID1P6 knockdown cells, but increased in PRELID1P6-overexpressed cells (Fig. 3G, H). We speculated that the observed effects might be attributable to proteasomal degradation because decreased hnRNPH1 levels were recovered by the proteasomal inhibitor MG-132 (Fig. 3I). In addition, PRELID1P6 knockdown in glioma cells decreased hnRNPH1 stabilization, shortening the half-life of hnRNPH1 (Fig. 3J). Moreover, we carried out immunoprecipitated assays in glioma cells using an anti-hnRNPH1 antibody and detected ubiquitin levels by western blotting. As expected, PRELID1P6 knockdown significantly increased the levels of ubiquitinated hnRNPH1, while PRELID1P6 overexpression decreased the levels of ubiquitinated hnRNPH1 (Fig. 3K, L). Since the proteasomal degradation typically takes place in the cytoplasm, we detected the expression of hnRNPH1 in nucleus and cytoplasm after PRELID1P6 knockdown, using western blot and IF assay. Result showed that knockdown of PRELID1P6 changed the cellular localization of hnRNPH1 from nucleus to cytoplasm (Fig. 3M, N). Collectively, these results indicated that the interaction of PRELID1P6 with hnRNPH1 in the cell nucleus inhibited the Ub degradation of hnRNPH1.

### HnRNPH1 is important for PRELID1P6-promoted glioma proliferation

**Fig. 4:**
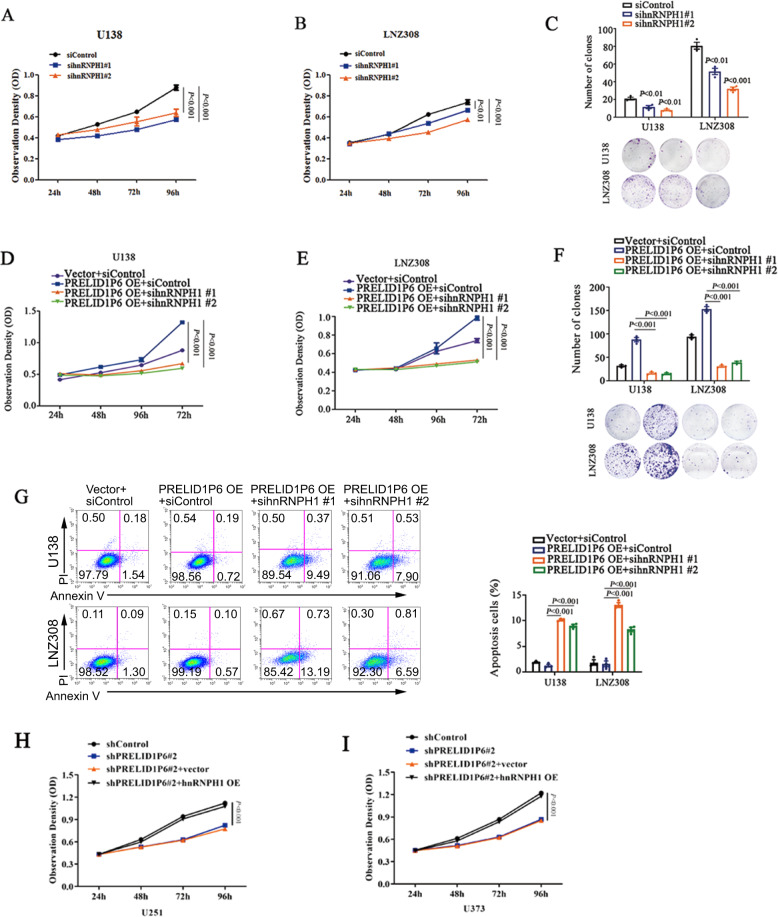
HnRNPH1 is important for PRELID1P6-promoted glioma proliferation. **A**, **B** Knockdown of hnRNPH1 inhibited U138 and LNZ308 glioma cell proliferation. **C** Knockdown of hnRNPH1 inhibited the clone formation ability of U138 and LNZ308 glioma cells. **D**, **E** Knockdown of hnRNPH1 reversed the growth -promotion effect of PRELID1P6 overexpression in U138 and LNZ308 cells. **F** Overexpression of PRELID1P6 promoted the U138 and LNZ308 cell clone formation ability, while knockdown of hnRNPH1 reversed the growth-promotion effect of PRELID1P6 overexpression. **G** Overexpression of PRELID1P6 decreased U138 and LNZ308 cell apoptosis, while knockdown of hnRNPH1 reversed the inhibited apoptosis effect of PRELID1P6 overexpression. **H**, **I** Knockdown of PRELID1P6 inhibited U251 and U373 glioma cells growth ability, while overexpression of hnRNPH1 reversed the growth-inhibition effect of PRELID1P6 knockdown.

Because we found that PRELID1P6 plays an oncogenic role in glioma and PRELID1P6 inhibited the ubiquitin-mediated degradation of hnRNPH1, we next investigated the impact of hnHNPH1 on glioma cell proliferation. We found that hnRNPH1 knockdown decreased glioma cell proliferation and clone formation ability (Fig. 4A‒C). Furthermore, small interfering RNA (siRNA) targeting hnRNPH1 inhibited the growth of both U138 and LNZ308 cells, which rescued PRELID1P6 overexpression-promoted growth and clone formation ability (Fig. 4D‒F). Knockdown of hnRNPH1-promoted glioma cell apoptosis, although PRELID1P6 was overexpressed (Fig. 4G). We also performed the rescue assay of proliferation after knockdown of PRELID1P6 with overexpression of hnRNPH1. Results showed that the growth of both U251 and U373 cells was inhibited with PRELID1P6 knockdown, while the inhibitory phenotypes were reversed after hnRNPH1 overexpression (Fig. 4H, I). Collectively, our results showed that hnRNPH1 is important for PRELID1P6-promoted glioma proliferation.

### Knockdown of PRELID1P6 inhibits Akt/mTOR phosphorylation in glioma

**Fig. 5:**
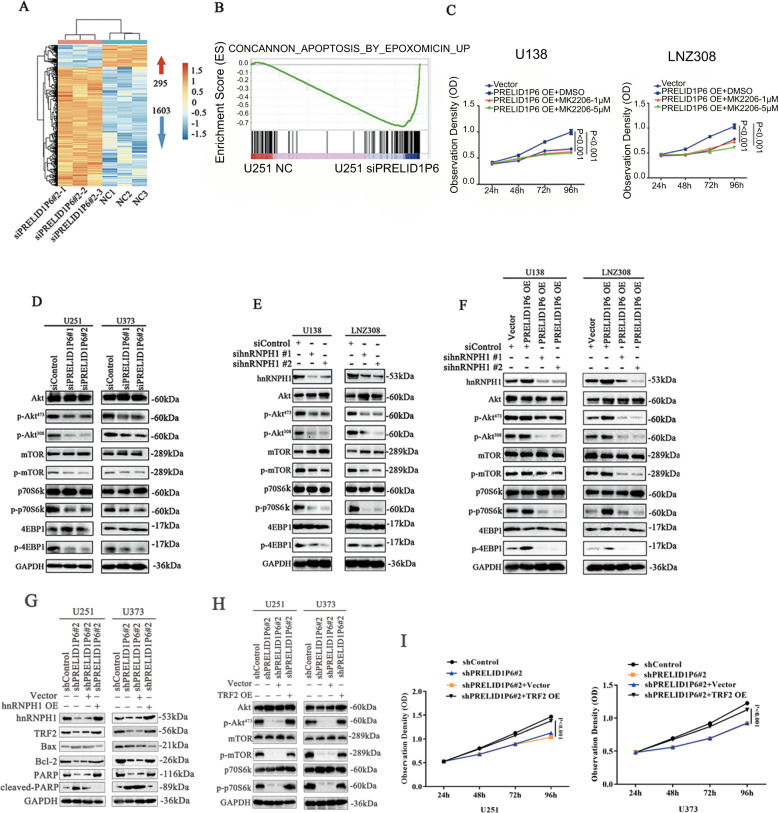
Knockdown of PRELID1P6 inhibits Akt/mTOR phosphorylation in glioma. **A** RNA sequencing showed that 1603 genes were downregulated and 295 genes were upregulated after PRELID1P6 knockdown in U251 cells. **B** GSEA pathway analysis showed that the knockdown of PRELID1P6 regulated the apoptosis signaling pathway. **C** PRELID1P6 overexpression-promoted growth of U138 and LNZ308 cells was reversed by MK2206 treatment at both 1 and 5 µM. **D** The phosphorylation levels of Akt/mTOR signaling pathway proteins were decreased in U251 (left) and U373 (right) cells with PRELID1P6 knockdown. **E** The phosphorylation levels of Akt/mTOR signaling pathway proteins were decreased in U251 (left) and U373 (right) cells with hnRNPH1 knockdown. **F** Knockdown of hnRNPH1 reversed the PRELID1P6 overexpression-activated phosphorylation levels of Akt/mTOR signaling pathway proteins. **G** PRELID1P6 knockdown decreased TRF2 and increased apoptosis-relative genes protein level, while overexpression of hnRNPH1 reverse the downregulation of TRF2 and upregulation of apoptosis-relative genes protein level. **H** PRELID1P6 knockdown decreased Akt, mTOR, and p70S6k phosphorylation level, while overexpression of TRF2 reverse downregulation of Akt, mTOR, and p70S6k phosphorylation level. **I** PRELID1P6 knockdown inhibited U251 and U373 glioma cells growth, while overexpression of TRF2 reverse growth-inhibiting effect of PRELID1P6 knockdown.

To determine the downstream signaling that was affected by PRELID1P6 knockdown in glioma cells, RNA-sequencing analyses were performed on U251 cells with PRELID1P6 knockdown or control. Knockdown of PRELID1P6 in U251 cells resulted in the upregulation of 1603 genes and downregulation of 295 genes (Fig. 5A). gene set enrichment analysis (GSEA) pathway analysis was performed on the differentially expressed genes from RNA-sequence data, and we found that the knockdown of PRELID1P6 regulated the apoptosis signaling pathway, which plays an important role in the cell proliferation (Fig. 5B). As reported previously, increased cell apoptosis may be caused by inactivated Akt [19]. We conducted an Akt phosphorylation inhibitor treatment assay using MK2206, and found that cell proliferation promoted by PRELID1P6 overexpression was dramatically inhibited by MK2206 in a dose-dependent manner in U138 and LNZ308 glioma cells (Fig. 5C). Furthermore, the phosphorylation levels of Akt/mTOR signaling pathway proteins were decreased after PRELID1P6 knockdown in glioma cells (Fig. 5D). Because hnRNPH1 is vital for PRELID1P6-promoted glioma proliferation, we also detected the phosphorylation levels of Akt/mTOR signaling pathway proteins after hnRNPH1 knockdown. The knockdown of hnRNPH1 deactivated the Akt/mTOR signaling pathway (Fig. 5E). In addition, the knockdown of hnRNPH1 attenuated the activation of the Akt/mTOR signaling pathway caused by PRELID1P6 overexpression (Fig. 5F). HnRNPH1 plays important role in RNA alternative splicing [20]. Some reports [21, 22] showed that hnRNPH1 could upregulate TRF2 by an alternative splicing effect. TRF2 is a component of the telomere nucleoprotein complex. It is present at telomeres in metaphase of the cell cycle, and plays a key role in the protective activity of telomeres. Reports showed that TRF2 played an important role in regulating Akt/mTOR signaling pathway and apoptosis process [23, 24]. Based on these researches, we detected the protein level of TRF2 after PRELID1P6 knockdown and performed a rescue assay. Results showed that knockdown of PRELID1P6 decreased the protein level of TRF2 and some key apoptosis-relative genes like Bax, Bcl-2, PARP, and Cleaved-PARP, while overexpression of hnRNPH1 reversed the downregulation of TRF2 protein (Fig. 5G). In addition, we performed a rescue assay using TRF2 overexpression plasmid. Results showed that knockdown of PRELID1P6 decreased Akt, mTOR, and p70S6k phosphorylation level and inhibited Akt/mTOR pathway, while TRF2 overexpression in shPRELID1P6#2 group reversed the downregulation of Akt, mTOR, and p70S6k phosphorylation level (Fig. 5H). Cell proliferation assay also showed that TRF2 overexpression could reverse the growth-inhibiting effect of PRELID1P6 knockdown (Fig. 5I), which mean that TRF2 was key downstream of PRELID1P6 and hnRNPH1, and play an important role in PRELID1P6-mediated Akt/mTOR signaling pathway activation. Collectively, we summarized that the knockdown of PRELID1P6 inhibited glioma progression via the hnHNPH1-Akt/mTOR pathway axis.

### PRELID1P6 is negatively regulated by miR-1825

**Fig. 6:**
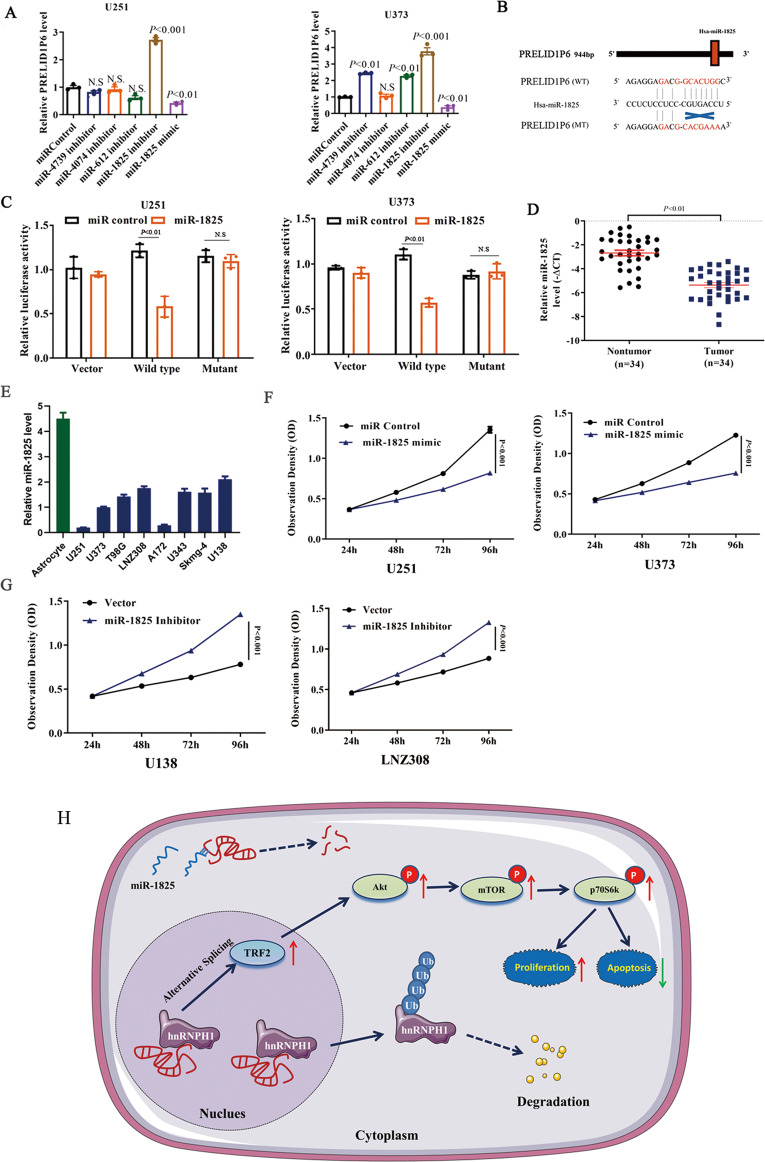
PRELID1P6 is negatively regulated by miR-1825. **A** The expression levels of PRELID1P6 were detected in U251 and U373 cells after transfection with miRNA inhibitors (miR-4739, miR-4074, miR-612, and miR-1825) and miR-1825 mimic, respectively. **B** Schematic of the interaction of miR-1825 with WT-PRELID1P6-luc or MT-PRELID1P6-luc. **C** The relative luciferase activities were detected by dual-luciferase reporter assays in U251 and U373 cells after cotransfection with miR-1825 mimics or miR-control and WT-PRELID1P6-luc, MT-PRELID1P6-luc or empty vector with pRL-CMV. **D** miR-1825 level was detected by qRT-PCR on 34 cases of paired fresh glioma and adjacent nontumorous brain tissues. **E** miR-1825 level was detected by qRT-PCR in eight glioma cell lines and normal immortalized astrocytes. **F** miR-1825 mimic impaired U251 and U373 glioma cells growth. **G** miR-1825 inhibitor promoted U138 and LNZ308 glioma cells growth. **H** A graphical abstract showing that the PRELID1P6 regulated proliferation and apoptosis by affecting hnRNPH1 stability and Akt/mTOR signaling pathway.

Accumulating evidence has shown that microRNAs can interact with noncoding genes and regulate noncoding gene expression levels [25]. The bioinformatics database RegRNA2.0 (http://regrna2.mbc.nctu.edu.tw/index.html) was used to identify candidate microRNAs that could target PRELID1P6. miR-4739, miR-4074, miR-612, and miR-1825 were found to possibly target PRELID1P6, but only downregulation of miR-1825 using the miR-1825 inhibitor (chemically synthesized inhibitors that targets miR-1825) increased the mRNA level of PRELID1P6. By contrast, the mRNA level of PRELID1P6 was decreased compared with the control sequence-treated group after miR-1825 mimics treatment (Fig. 6A). Furthermore, the dual-luciferase reporter assay was performed to verify whether PRELID1P6 is a direct target of miR-1825. We established two constructs, wild-type (WT)-PRELID1P6 3′-UTR and mutant (MT)-PRELID1P6 3′-UTR. Empty luciferase reporter constructs served as the control. A model describing the interaction between miR-1825 and PRELID1P6, as well as the establishment of the WT-PRELID1P6 3ʹ-UTR and MT-PRELID1P6 3ʹ-UTR constructs, is shown in Fig. 6B. The relative luciferase activity in U251 and U373 cells appeared to significantly diminish after the cotransfection of miR-1825 mimics (chemically synthesized miR-1825) and WT-PRELID1P6-luc, while the luciferase activity was not affected in the empty vector group or MT-PRELID1P6-luc group (Fig. 6C). We detected the expression of miR-1825 on 34 cases of paired fresh glioma and adjacent nontumorous brain tissues, results showed that miR-1825 was significantly downregulated in glioma tissues compared to that in nontumorous brain tissues (Fig. 6D). We also detected the expression of miR-1825 on eight glioma cells and an astrocyte cell line. MiR-1825 were upregulated in U138 and LNZ308, but downregulated in U251 and U373, while maintained at highest level in astrocytes (Fig. 6E). Furthermore, the proliferation of U251 and U373 were impaired by the treatment of miR-1825 mimic, which was even promoted in U138 and LNZ308 treated by inhibitor of miR-1825 (Fig. 6F, G). Collectively, these findings support that PRELID1P6 is negatively regulated by miR-1825.

## Discussion

In recent years, the roles of pseudogenes were reported to be associated with various human diseases, including cancer, and have attracted much attention [26–28]. Several pseudogenes are differentially expressed in glioma tissues and are implicated in the biological processes related to tumorigenesis and drug resistance to chemotherapy [17, 29]. Until now, no report has investigated the function and mechanism of the pseudogene PRELID1P6 in glioma. These findings shed new light on the importance of the orchestrated interactions among pseudogenes, microRNAs, and proteins in tumorigenesis, and may be used for precision therapy in glioma patients.

Multivariate Cox regression analysis showed that PRELID1P6 expression and IDH mutation were both independent prognostic factors in glioma. We explored the clinical significance of PRELID1P6 in glioma and found that high expression of PRELID1P6 was related to a poor prognosis. Functional assays showed that PRELID1P6-promoted glioma cell proliferation and inhibited cell apoptosis, suggesting that PRELID1P6 promotes glioma progression. Taken together, our findings indicate the pro-proliferation roles of PRELID1P6 in glioma.

Coexpression analyses and rescue experiments confirmed the interaction between PRELID1P6 and hnRNPH1, suggesting that hnRNPH1 is a key mediator for PRELID1P6 in promoting cell proliferation in glioma. Apart from the roles as antisense RNA or endogenous small-interference RNA, pseudogenes can also function as endogenous competitors for miRNA, RBPs, or the translational machinery [14]. HnRNPs represent a large family of RBPs that contribute to multiple aspects of nucleic acid metabolism, including alternative splicing, mRNA stabilization, and transcriptional and translational regulation [30]. The best-known function of hnRNP F and H, in combination with other hnRNPs, is the regulation of alternative splicing [31]. Jinmu Deng et al. showed that hnRNP A2/B1 is an oncogene in glioma [32]. However, little is known about the function and mechanism of hnRNPH1 in glioma. In the present study, we found that hnRNPH1-promoted glioma proliferation and interacted with PRELID1P6. Further investigation revealed that PRELID1P6 inhibited the Ub of hnRNPH1. Protein Ub is a highly conserved modification that controls various cellular processes and is covalently attached to protein substrates by a cascade of enzymatic reactions [33]. The regulation of ubiquitin signaling, which involves the ability of ubiquitin to form different types of chains on itself, led to distinct biological outcomes [34, 35]. Since proteasomal degradation typically takes place in the cytoplasm, our result also showed that knockdown of PRELID1P6 change the cellular localization of hnRNPH1 from nucleus to cytoplasm, which promoted the Ub degradation of hnRNPH1.

RNA sequencing performed after PRELID1P6 knockdown in glioma cells and GSEA based on differentially expressed genes suggested that the knockdown of PRELID1P6 enriched apoptosis-associated genes. Because reports have shown that increased cell apoptosis may be caused by inactivated Akt [19], we focused on the Akt/mTOR signaling pathway. In our study, western blotting showed that the knockdown of PRELID1P6 decreased the phosphorylation of key Akt/mTOR signaling pathway proteins in an hnRNPH1-dependent manner. In the past decade, many studies have reported that lncRNAs directly interacts with mTOR complex, and functions upstream or downstream of mTOR signaling [36]. In addition, hnRNPH1 acts as a splicing factor regulated by Akt [37]. In this study, we showed that PRELID1P6 increased TRF2 expression through hnRNPH1-mediated alternative splicing effect and subsequently activated the Akt/mTOR signaling pathway. The Akt/mTOR signaling pathway regulates cell proliferation and self-renewal, promoting glioma proliferation [38, 39].

It has been documented that noncoding gene levels are regulated by microRNAs [25]. Pseudogenes, as noncoding genes, are also targeted by microRNAs [40]. Our results showed that miR-1825 targets PRELID1P6 and downregulates PRELID1P6 expression. miR-1825 was previously reported to play an important role in the development of human glioma, regulating apoptosis, cell proliferation, and invasion [41]. Pseudogenes are reported as an ideal target because they are widely involved in multiple processes [42].

In this study, we identified that PRELID1P6, which was negatively regulated by miR-1825, bound to hnRNPH1 to prevent its Ub degradation in cell nucleus, which subsequently increased TRF2 expression by alternative splicing effect, activated the Akt/mTOR pathway, and promoted glioma cells proliferation and inhibited cell apoptosis. (Fig. 6H). These finding shed new light on the importance of the orchestrated interactions between pseudogenes, microRNAs, and proteins in tumorigenesis, providing a greater possibility for precision therapy in glioma patients.

## Materials and methods

### Patients and data collection

Surgically resected specimens from 222 cases were collected between January 2000 and December 2015 for tissue microarray analysis. Another 34 cases of paired fresh glioma and adjacent nontumorous brain tissues were collected from patients at the time of surgical resection. The use of human tissues was approved by the SYSUCC Institute Research Ethics Committee, and informed consent was obtained from the patients or their relatives.

### In situ hybridization and immunohistochemical staining

RNA ISH was performed on glioma tissue microarray using an RNA In Situ Hybridization Kit (Exonbio Lab, Guangzhou, China), according to the manufacturer’s instructions. The expression levels of PRELID1P6 in glioma were scored as the proportion of the immunopositive staining area (0‒100%) multiplied by the intensity of staining (0, negative; 1, weak; 2, moderate; and 3, intense). The scores were independently determined by two pathologists (SX and YZ). The median IHC score was chosen as the cutoff value for defining high and low expression of PRELID1P6.

### Cell lines

Human glioma cell lines U251, U373, T98G, U138, LNZ308, A172, U343, and Skmg-4, 293T cells, and the human immortalized normal astrocyte cell line were used for this study. All the cell lines were tested for mycoplasma and were found to be free from infection. The U251, U373, T98G, LNZ308, A172, U343, and Skmg-4 cells and 293T cells were maintained in Dulbecco’s Modified Eagle Medium (DMEM) medium supplemented with 10% fetal bovine serum (FBS).

### Lentivirus production and transduction

The lentiviral vector system was purchased from OBiO Technology (Shanghai, China). Full-length PRELID1P6 was cloned into the H145 pLenti-EF1a-EGFP-F2A-Puro-CMV-MCS vector. A lentiviral vector encoding the human PRELID1P6 was designated as PRELID1P6OE, and the empty vectors served as a negative control (Vector). Lentiviral constructs containing the siRNA sequence (Table S2) targeting human PRELID1P6 were inserted into the pLKD-CMV-G&PR-U6-shRNA vector and were used to establish cell lines constitutively repressing PRELID1P6 (named siPRELID1P6#1 and siPRELID1P6#2). Glioma cells were infected with concentrated virus in the presence of polybrene (8 μg/mL; Sigma-Aldrich, Milwaukee, WI) and were selected with puromycin (8 μg/mL).

### RNA interference

siRNA oligonucleotides targeting PRELID1P6, hnRNPH1, and control siRNA (Table S2) were purchased from OBio. Transfections with siRNA (75 nM) were performed using Lipofectamine 3000 (Invitrogen, Carlsbad, CA, USA).

### RNA isolation and qRT-PCR

The total RNA of glioma samples and cell lines was isolated using TRIZOL reagent (Invitrogen) followed by reverse transcription using the RevertAid First Strand cDNA Synthesis Kit (Thermo Fisher, Rockford, USA). qRT-PCR was performed to determine the relative RNA level using the SYBR Green method in the Bio-Rad CFX96 Real-Time PCR System (Bio-Rad Laboratories, Inc., Hercules, CA). GAPDH and U6 small nuclear RNA were employed as endogenous controls. The relative RNA expression levels were calculated using the comparative Ct formula. All the primer sequences used in this study are listed in Table S2.

### Dual-luciferase reporter assay

The 3′-UTR of PRELID1P6 (GCACTGGC) and the MT form (CACGAAAA) were separately subcloned into a pEZX-MT06 vector that contained the firefly luciferase gene (OBio) to establish two constructs, the WT-PRELID1P6-luc and MT-PRELID1P6-luc, respectively. The pRL-CMV vector containing the Renilla luciferase gene served as an internal control. Overexpression constructs, including the miR-1825 mimics and control, were cotransfected with WT-PRELID1P6-luc and MT-PRELID1P6-luc into U251 and U373 cells, respectively. The luciferase activities were detected 24 h after transfection using the Dual-Luciferase Reporter Assay System (Promega, Madison, WI), according to the manufacturer’s instructions.

### Subcellular fractionation analysis

Subcellular isolation of RNAs in U251 and U373 cells was conducted using the Cytoplasmic and Nuclear RNA Purification Kit (Norgenbiotek, Ontario, Canada) according to the manufacturer’s instructions. The cytoplasmic and nuclear fractions were determined by qRT‒PCR.

### Cell proliferation, cell cycle, apoptosis, clone forming assay, and sphere formation assay

The cells were seeded in 96-well plates, with each well containing 3000 cells. The cell viability was measured using the Cell Counting Kit-8 assay (Dojindo, Japan). Each experiment with six replicates was repeated three times. For cell cycle analysis, the cells were collected and fixed in 70% ethanol overnight at 4 °C. Single-cell suspensions were labeled with 50 μg/mL of propidium iodide (KeyGEN, Nanjing, China) and analyzed by flow cytometry (Beckman Coulter, Indianapolis, USA). For apoptosis, the cells were stained with Annexin V-FITC or Annexin V-APC (BD Biosciences, New Jersey, USA) and propidium iodide, and the percentage of apoptotic cells was determined by flow cytometry. For the sphere formation assay, 300 cells were seeded in ultralow attachment six-well plates in DMEM/F12, supplemented with 20 ng/mL of EGF, 20 ng/mL of bFGF, and 2% B27. Spheres with >50 cells were counted after culture for 14 days. For clone formation assays, the cells were seeded into six-well plates with DMEM medium supplemented with 10% FBS at a density of 300 cells per well. After culture for 14 days, the clones were fixed with 4% paraformaldehyde and stained with crystal violet. Clones with >50 cells were counted.

### Xenograft growth of glioma cells in mice

Female BALB/c nude mice, aged 4–5 weeks, were purchased from the Model Animal Research Center of Nanjing University (Nanjing, Jiangsu, China). The mice (five in each group) were injected with 200 μL of a cell suspension containing 2 × 10^6^ cells in their subcutaneous tissue. The tumor volumes were measured every other day, and the sizes were calculated according to the following formula: volume = length × width^2^ × 0.5. The animal experiments were approved by the Ethics Committee of SYSUCC.

### RNA pull-down assay and mass spectrometry

The RNA pull-down assay was performed using the Pierce Magnetic RNA–Protein Pull-down kit (Thermo Fisher), according to the manufacturer’s instructions. Briefly, sense and antisense RNAs were transcribed in vitro by T7 RNA polymerase using the MEGAscript kit (Ambion, Carlsbad, CA) and were labeled using the Pierce RNA 3′ Desthiobiotinylation Kit (Thermo Fisher). Next, 50 pmol of biotin-labeled RNA was used for RNA pull-down assays. The eluted proteins were detected by western blot analysis and mass spectrometry, using the MALDI-TOF‒MS instrument (Bruker Daltonics, Billerica, USA).

### RNA immunoprecipitation assay

RIP assays were performed using the RNA-Binding Protein Immunoprecipitation Kit (Millipore, Burlington, MA), according to the manufacturer’s instructions. Five micrograms of rabbit anti-hnRNPH1 (Abcam) antibody was used to perform RIP. The enrichment of PRELID1P6 was determined by qRT‒PCR.

### Cycloheximide (CHX) chase assay

To observe the degradation process of proteins, CHX (MP Biomedicals, Santa Ana, CA) was introduced to the cell culture medium at a final concentration of 100 ng/mL. Total protein was extracted at 0, 15, 30, 60, 120, and 240 min after CHX treatment and analyzed by western blotting.

### In vitro ubiquitination assay

Cells were treated with MG-132 (5 μM; Selleck Chemicals, Houston, USA) for 24 h. Cell lysates were then prepared and subjected to immunoprecipitation with rabbit anti-hnRNPH1 antibody (Abcam). The elution was assessed by western blotting with the mouse anti-ubiquitin antibody.

### Colocation of PRELID1P6 and hnRNHP1

RNA FISH and IF staining were combined to detect the colocalization of PRELID1P6 and hnRNPH1 in cells. Hybridization with the PRELID1P6 RNA probe (site of probe targeting PRELID1P6: 457‒944; Exonbio Lab) was first performed at 37 °C for 4 h, followed by the incubation with rabbit anti-hnRNPH1 antibody (Abcam) overnight at 4 °C. After secondary antibody incubation and DAPI (Exonbio) staining, the obtained images were captured using a confocal microscope (Olympus FV1000, Tokyo, Japan).

### RNA sequencing and GSEA

RNA was extracted from triplicate samples of siPRELID1P6 U251 cells and the corresponding U251 control cells. RNA sequencing was performed by Novogene Company (Guangzhou, China) using the PE150 platform. GSEA was used to analyze the data.

### Antibodies and western blot assays

Western blot assays were performed according to standard protocol [43]. Information regarding the antibodies is listed in Table S3.

### Statistical analysis

Statistical analyses were performed using the Statistical Product and Service Solutions software, version 25.0 (SPSS, Chicago, IL). Paired Student’s *t* test was used to analyze the PRELID1P6 expression level in glioma specimens. Kaplan–Meier plots and the log-rank test were used for the overall survival analysis. Independent Student’s *t* test was used to compare the cell growth rate, clone formation, sphere formation, and tumor growth rate. The results with *P* < 0.05 were considered statistically significant.

## Supplementary information

Supplementary Figure 1

Supplementary Figure 2

Supplementary figure 3

Supplementary figure 4

Supplementary material

## Data Availability

The raw data of this paper have been uploaded onto the Research Data Deposit (RDD) with an RDD number of RDDB2020000835.

## References

[1] Xu W, Li T, Gao L, Zheng J, Shao A, Zhang J (2017). Efficacy and safety of long-term therapy for high-grade glioma with temozolomide: a meta-analysis. Oncotarget..

[2] Sizoo EM, Braam L, Postma TJ, Pasman HR, Heimans JJ, Klein M (2010). Symptoms and problems in the end-of-life phase of high-grade glioma patients. Neuro-Oncol.

[3] Barth RF, Zhang Z, Liu T (2018). A realistic appraisal of boron neutron capture therapy as a cancer treatment modality. Cancer Commun.

[4] Ostrom QT, Gittleman H, Truitt G, Boscia A, Kruchko C, Barnholtz-Sloan JS (2018). CBTRUS statistical report: primary brain and other central nervous system tumors diagnosed in the United States in 2011–2015. Neuro-Oncol.

[5] Lander ES, Linton LM, Birren B, Nusbaum C, Zody MC, Baldwin J (2001). Initial sequencing and analysis of the human genome. Nature..

[6] Khalil AM, Guttman M, Huarte M, Garber M, Raj A, Rivea Morales D (2009). Many human large intergenic noncoding RNAs associate with chromatin-modifying complexes and affect gene expression. Proc Natl Acad Sci USA.

[7] Rinn JL, Kertesz M, Wang JK, Squazzo SL, Xu X, Brugmann SA (2007). Functional demarcation of active and silent chromatin domains in human HOX loci by noncoding RNAs. Cell..

[8] Nagano T, Mitchell JA, Sanz LA, Pauler FM, Ferguson-Smith AC, Feil R (2008). The air noncoding RNA epigenetically silences transcription by targeting G9a to chromatin. Science..

[9] Shan K, Jiang Q, Wang XQ, Wang YN, Yang H, Yao MD (2016). Role of long non-coding RNA-RNCR3 in atherosclerosis-related vascular dysfunction. Cell Death Dis.

[10] Yang N, Chen J, Zhang H, Wang X, Yao H, Peng Y (2017). LncRNA OIP5-AS1 loss-induced microRNA-410 accumulation regulates cell proliferation and apoptosis by targeting KLF10 via activating PTEN/PI3K/AKT pathway in multiple myeloma. Cell Death Dis.

[11] Bracken CP, Scott HS, Goodall GJ (2016). A network-biology perspective of microRNA function and dysfunction in cancer. Nat Rev Genet.

[12] Shan Y, Ma J, Pan Y, Hu J, Liu B, Jia L (2018). LncRNA SNHG7 sponges miR-216b to promote proliferation and liver metastasis of colorectal cancer through upregulating GALNT1. Cell Death Dis.

[13] Jacq C, Miller JR, Brownlee GG (1977). A pseudogene structure in 5S DNA of *Xenopus laevis*. Cell..

[14] Xiao-Jie L, Ai-Mei G, Li-Juan J, Jiang X (2015). Pseudogene in cancer: real functions and promising signature. J Med Genet.

[15] Wang L, Guo ZY, Zhang R, Xin B, Chen R, Zhao J (2013). Pseudogene OCT4-pg4 functions as a natural micro RNA sponge to regulate OCT4 expression by competing for miR-145 in hepatocellular carcinoma. Carcinogenesis..

[16] Karreth FA, Reschke M, Ruocco A, Ng C, Chapuy B, Leopold V (2015). The BRAF pseudogene functions as a competitive endogenous RNA and induces lymphoma in vivo. Cell..

[17] Zhang Y, Li Y, Wang J, Lei P (2018). Long noncoding RNA ferritin heavy polypeptide 1 pseudogene 3 controls glioma cell proliferation and apoptosis via regulation of the microRNA2245p/tumor protein D52 axis. Mol Med Rep.

[18] Guttman M, Donaghey J, Carey BW, Garber M, Grenier JK, Munson G (2011). lincRNAs act in the circuitry controlling pluripotency and differentiation. Nature..

[19] Edwards LA, Thiessen B, Dragowska WH, Daynard T, Bally MB, Dedhar S (2005). Inhibition of ILK in PTEN-mutant human glioblastomas inhibits PKB/Akt activation, induces apoptosis, and delays tumor growth. Oncogene.

[20] Kedzierska H, Piekielko-Witkowska A (2017). Splicing factors of SR and hnRNP families as regulators of apoptosis in cancer. Cancer Lett.

[21] Grammatikakis I, Zhang P, Panda AC, Kim J, Maudsley S, Abdelmohsen K (2016). Alternative splicing of neuronal differentiation factor TRF2 regulated by HNRNPH1/H2. Cell Rep.

[22] Grammatikakis I, Zhang P, Mattson MP, Gorospe M (2016). The long and the short of TRF2 in neurogenesis. Cell Cycle..

[23] Wang F, Sheng JF, Cai L, Xu Y, Liao H, Tao ZZ (2018). The telomerase and alternative lengthening of telomeres mechanisms regulate laryngeal cancer cell apoptosis via the PI3K/Akt pathway. ORL J Otorhinolaryngol Relat Spec..

[24] Werner C, Hanhoun M, Widmann T, Kazakov A, Semenov A, Poss J (2008). Effects of physical exercise on myocardial telomere-regulating proteins, survival pathways, and apoptosis. J Am Coll Cardiol.

[25] Chen X, Liang H, Zhang CY, Zen K (2012). miRNA regulates noncoding RNA: a noncanonical function model. Trends Biochem Sci.

[26] Hu X, Yang L, Mo YY. Role of pseudogenes in tumorigenesis. Cancers (Basel). 2018;10:256. 10.3390/cancers10080256 PMC6115995 30071685

[27] Lian Y, Yang J, Lian Y, Xiao C, Hu X, Xu H (2018). DUXAP8, a pseudogene derived lncRNA, promotes growth of pancreatic carcinoma cells by epigenetically silencing CDKN1A and KLF2. Cancer Commun..

[28] Chen CL, Tseng YW, Wu JC, Chen GY, Lin KC, Hwang SM (2015). Suppression of hepatocellular carcinoma by baculovirus-mediated expression of long non-coding RNA PTENP1 and MicroRNA regulation. Biomaterials..

[29] Rynkeviciene R, Simiene J, Strainiene E, Stankevicius V, Usinskiene J, Miseikyte Kaubriene E, et al. Non-coding RNAs in glioma. Cancers (Basel). 2018;11:17. 10.3390/cancers11010017 PMC6356972 30583549

[30] Geuens T, Bouhy D, Timmerman V (2016). The hnRNP family: insights into their role in health and disease. Hum Genet.

[31] Gautrey H, Jackson C, Dittrich AL, Browell D, Lennard T, Tyson-Capper A (2015). SRSF3 and hnRNP H1 regulate a splicing hotspot of HER2 in breast cancer cells. RNA Biol..

[32] Deng J, Chen S, Wang F, Zhao H, Xie Z, Xu Z (2016). Effects of hnRNP A2/B1 knockdown on inhibition of glioblastoma cell invasion, growth and survival. Mol Neurobiol..

[33] Kerscher O, Felberbaum R, Hochstrasser M (2006). Modification of proteins by ubiquitin and ubiquitin-like proteins. Annu Rev Cell Dev Biol.

[34] Xu P, Duong DM, Seyfried NT, Cheng D, Xie Y, Robert J (2009). Quantitative proteomics reveals the function of unconventional ubiquitin chains in proteasomal degradation. Cell..

[35] Grice GL, Nathan JA (2016). The recognition of ubiquitinated proteins by the proteasome. Cell Mol Life Sci.

[36] Aboudehen K. Regulation of mTOR signaling by long non-coding RNA. Biochim Biophys Acta Gene Regul Mech. 1863;2019:194449. 10.1016/j.bbagrm.2019.194449 PMC7125019 31751821

[37] da Silva MR, Moreira GA, Goncalves da Silva RA, de Almeida Alves Barbosa E, Pais Siqueira R, Teixera RR (2015). Splicing regulators and their roles in cancer biology and therapy. Biomed Res Int..

[38] Li X, Wu C, Chen N, Gu H, Yen A, Cao L (2016). PI3K/Akt/mTOR signaling pathway and targeted therapy for glioblastoma. Oncotarget..

[39] Sarkar S (2013). Regulation of autophagy by mTOR-dependent and mTOR-independent pathways: autophagy dysfunction in neurodegenerative diseases and therapeutic application of autophagy enhancers. Biochem Soc Trans.

[40] Poliseno L, Salmena L, Zhang J, Carver B, Haveman WJ, Pandolfi PP (2010). A coding-independent function of gene and pseudogene mRNAs regulates tumour biology. Nature..

[41] Xing W, Zeng C (2017). A novel serum microRNA-based identification and classification biomarker of human glioma. Tumour Biol.

[42] Chen X, Wan L, Wang W, Xi WJ, Yang AG, Wang T (2020). Re-recognition of pseudogenes: from molecular to clinical applications. Theranostics.

[43] Shen D, Guo CC, Wang J, Qiu ZK, Sai K, Yang QY (2015). Interferon-alpha/beta enhances temozolomide activity against MGMT-positive glioma stem-like cells. Oncol Rep.

