# Supplementary figures and images for: The pseudogene PRELID1P6 promotes glioma progression via the hnHNPH1-Akt/mTOR axis
Source: Oncogene. 2021 Jun 9;40(26):4453–67. doi: 10.1038/s41388-021-01854-x (PMC8249232; doi:10.1038/s41388-021-01854-x)

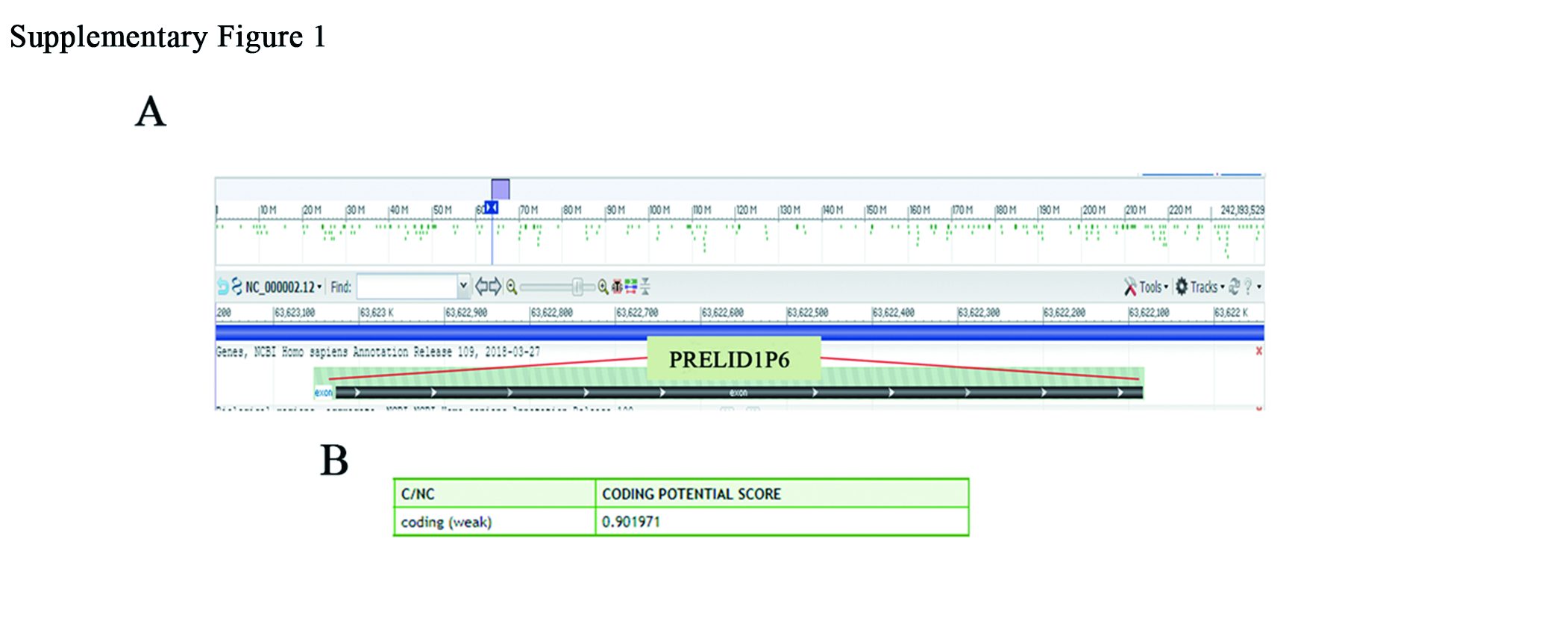

Supplement: Supplementary file 1 — Supplementary Figure 1 [file 41388_2021_1854_MOESM1_ESM.tif]

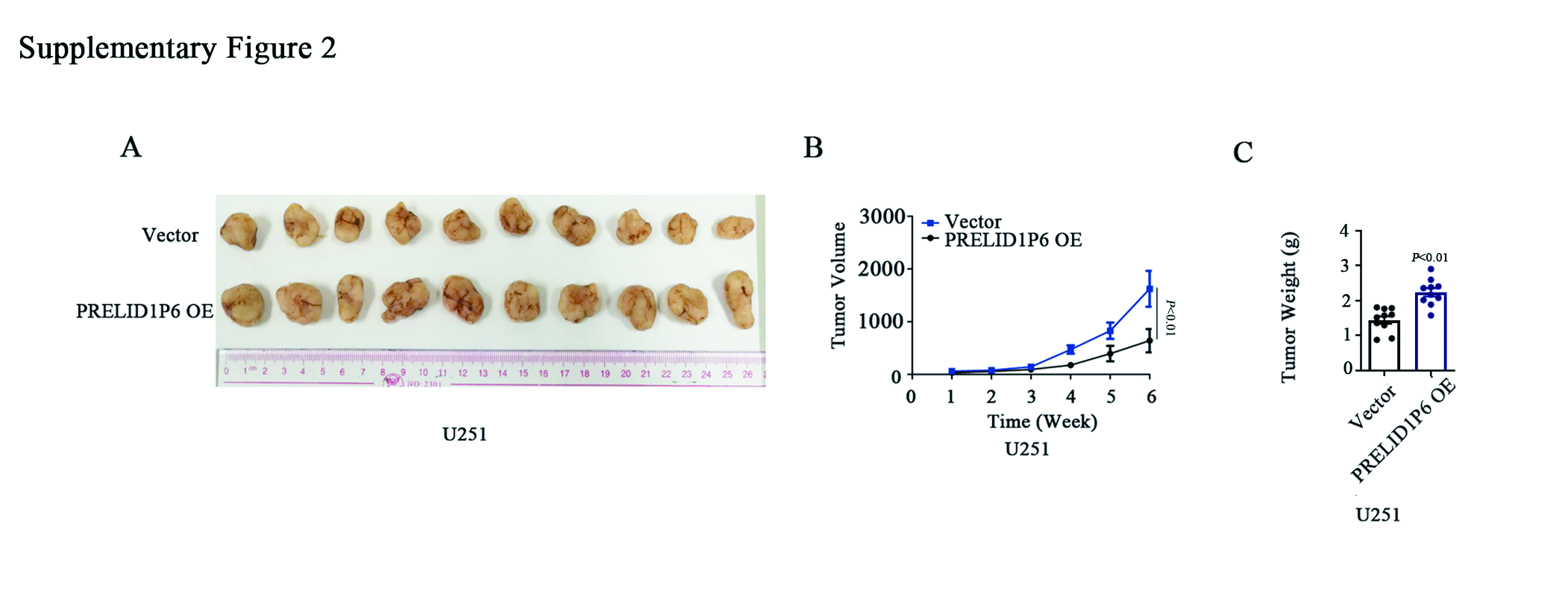

Supplement: Supplementary file 2 — Supplementary Figure 2 [file 41388_2021_1854_MOESM2_ESM.tif]

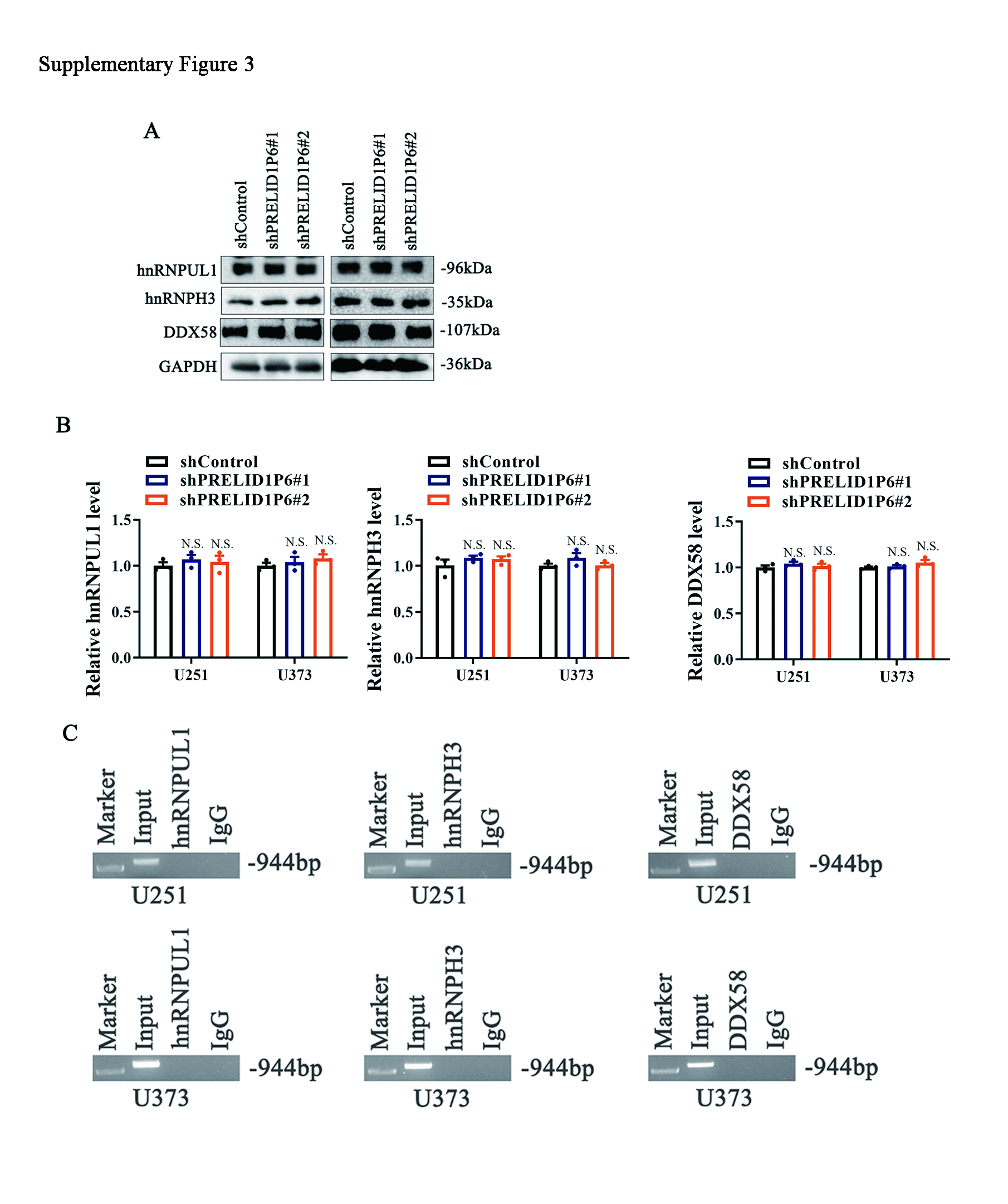

Supplement: Supplementary file 3 — Supplementary figure 3 [file 41388_2021_1854_MOESM3_ESM.tif]

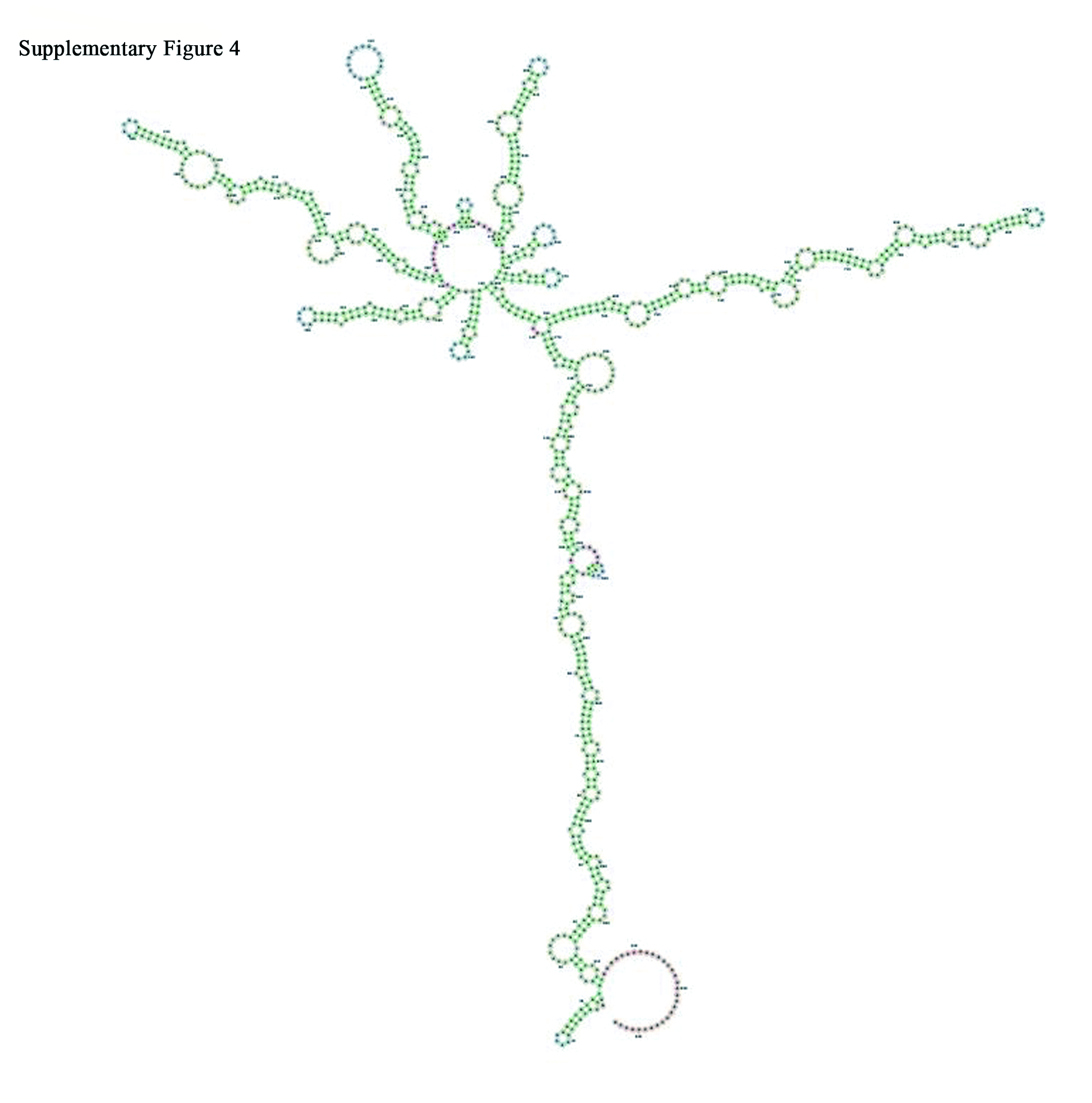

Supplement: Supplementary file 4 — Supplementary figure 4 [file 41388_2021_1854_MOESM4_ESM.tif]
