# Supplementary material for: The pseudogene PRELID1P6 promotes glioma progression via the hnHNPH1-Akt/mTOR axis
Source: Oncogene. 2021 Jun 9;40(26):4453–67. doi: 10.1038/s41388-021-01854-x (PMC8249232; doi:10.1038/s41388-021-01854-x)
Supplement: Supplementary file 5 — Supplementary material [file 41388_2021_1854_MOESM5_ESM.pdf]

Supplementary figure and table

Supplementary figure 1. The characteristics and expression of PRELID1P6

(A) Location of PRELID1P6, chromosome 2p15. (B) Prediction of protein-coding ability of PRELID1P6 by Coding Potential Calculator.

Supplementary figure 2. Overexpression of PRELID1P6 significantly increased the *in vitro* and *in vivo* proliferation of glioma cells.

(A, B, C). Xenograft tumor formation of U251 cells with PRELID1P6 overexpression in nude mice. Growth rates, tumor weights were significantly increased compared to the control group.

Supplementary figure 3. PRELID1P6 did not interact with other RNA binding protein identified by MS.

(A) The protein level of hnRNPUL1, hnRNPH3 and DDX58 did not change after PRELID1P6 knockdown in U251 and U373 glioma cells. (B) The mRNA level of hnRNPUL1, hnRNPH3 and DDX58 did not change after PRELID1P6 knockdown in U251 and U373 glioma cells. (C) IP assay using hnRNPUL1, hnRNPH3 and DDX58 antibody did not pulldown PRELID1P6.

Supplementary figure 4. The secondary structure of PRELID1P6

The secondary structures as predicted by RNAfold

Supplementary Table 1. List of PRELID1P6-interacting proteins identified by Mass Spectrometry

Gene name	Protein name	Mass	#1 score	#2 score	#3 score
HNRNPH1	Heterogeneous nuclear ribonucleoprotein H	49484	280	201	218
DDX58	Probable ATP-dependent RNA helicase DDX58	108014	180	70	156
ETFA	Electron transfer flavoprotein subunit alpha, mitochondrial	35400	127	34	61
SEPT11	Septin-11	49652	118	89	83
HNRNPUL1	Heterogeneous nuclear ribonucleoprotein U-like protein 1	96250	95	62	36
HPRT1	Hypoxanthine-guanine phosphoribosyltransferase	24792	91	32	55
RAB2A	Ras-related protein Rab-2A	23702	90	76	87
HNRNPH3	Heterogeneous nuclear ribonucleoprotein H3	36960	89	64	35
RRM2	Ribonucleoside-diphosphatered uctase subunit M2	45134	86	51	51
ACTR3	Actin-related protein 3	47797	80	42	34

Supplementary Table 2. Sequences of primers, shRNA and siRNA used for in this study

Sequences of primers and probes for qPCR
PRELID1P6 F: 5'- AACAGCGGCTGGACTGAAAT-3'
PRELID1P6 R: 5'- TCACGTTGCTTTTGAACTGGG-3'
U6 F: 5'-CTCGCTTCGGCAGCACA-3'
U6 R: 5'-AACGCTTCACGAATTTGCGT-3'
hGAPDH F: 5'- ACCACAGTCCATGCCATCAC -3'
hGAPDH R: 5'- CACCACCCTGTTGCTGTAGCC -3'
hnRNPH1 F: 5'-ATTCAAAATGGGGCTCAAGGTAT -3'
hnRNPH1 R: 5'-GTGTCAGGACTATTTGGACCAG-3'
siPRELID1P6#1: CTGGGCCAAGCAACTATTT
siPRELID1P6#2: GAATGCCACGCTGGGCCAA
sihnRNPH1#1: GGAGCTGGCTTTGAGAGGA
sihnRNPH1#2: GCATACTGGTCCAAATAGT
hAkt F: AGCGACGTGGCTATTGTGAAG
hAkt R: GCCATCATTCTTGAGGAGGAAGT
hmTOR F: ATGCTTGGAACCGGACCTG
hmTOR R: TCTTGACTCATCTCTCGGAGTT
hp70S6k F: CGGGACGGCTTTTACCCAG
hp70S6k R: TTTCTCACAATGTTCCATGCCA

Supplementary Table 3. List of antibodies used in western blot, immunoprecipitation analysis

Antibody	Vendor	Catalog No	Application
Rabbit anti-hnRNPH1	Abcam	Ab10374	WB,1:5000; IP, 6μg/mg of lysate
Rabbit anti-human Atk	Cell Signaling	4691	WB, 1:1000
Rabbit anti-human Phospho-Akt (Ser473)	Cell Signaling	4060	WB, 1:1000
Rabbit anti-human Phospho-Akt (Thr308)	Cell Signaling	13038	WB, 1:1000
Rabbit anti-human mTOR	Cell Signaling	2983	WB, 1:1000
Rabbit anti-human Phospho-mTOR (Ser2448)	Cell Signaling	5536	WB, 1:1000
Rabbit anti-human p70 S6K	Cell Signaling	2708	WB, 1:1000
Rabbit anti-human Phospho-p70 S6K(Ser371)	Cell Signaling	9208	WB, 1:1000
Rabbit anti-human 4E-BP1	Cell Signaling	9644	WB, 1:1000
Rabbit anti-human Phospho-4E-BP1(Ser65)	Cell Signaling	9451	WB, 1:1000
Mouse anti-human Ubiquitin	Cell Signaling	3936	WB, 1:1000
Mouse anti-human GAPDH	Proteintech	60004-1	WB, 1:1000
Mouse anti-human TRF2	Santa scruze	sc-271710	WB, 1:1000
Mouse anti-human hnRNPUL1	Santa scruze	sc-393975	WB, 1:1000
Mouse anti-human hnRNPH3	Santa scruze	sc-376416	WB, 1:1000
Rabbit anti-human DDX58	Abcam	ab180675	WB, 1:1000
